# Airway Management of Esophageal Atresia and Tracheoesophageal Fistula Combined with Anal Atresia

**DOI:** 10.1155/2022/3775140

**Published:** 2022-09-05

**Authors:** Jieshu Zhou, Hao Li, Xuemei Lin

**Affiliations:** Department of Anesthesiology, West China Second University Hospital, Sichuan University, No. 20, Section 3, South of Renmin Road, Chengdu 610041, Sichuan, China

## Abstract

Esophageal atresia (EA) associated with tracheoesophageal fistula (TEF) is a common congenital airway anomaly and may be associated with other gastrointestinal abnormalities. Neonates with EA/TEF are at high risk of gastrointestinal distension due to the shunting of air via the fistula, leading to progressive diaphragmatic elevation and regurgitation of the gastrointestinal contents. EA/TEF associated with anal atresia in a neonate makes airway management even more challenging particularly when managed for the repair of TEF through thoracotomy. Here, we report a case where we succeeded in conducting the flexible bronchoscopy insertion through a laryngeal mask to block the fistula by bronchial blocker under spontaneous breathing.

## 1. Introduction

EA/TEF is one of the common congenital airway anomalies with an incidence of 1 in 4100 live births, and it can also occur in association with other congenital disabilities [1]. EA/TEF with anal atresia is not uncommon, with an incidence of 14% [2]. Anal atresia when associated with EA/TEF can further aggravate the gastrointestinal distension causing progressive diaphragmatic elevation leading to respiratory failure. Thus, the airway management of a neonate with EA/TEF and anal atresia is challenging for the anesthesiologist.

## 2. Case Presentation

**Figure 1:**
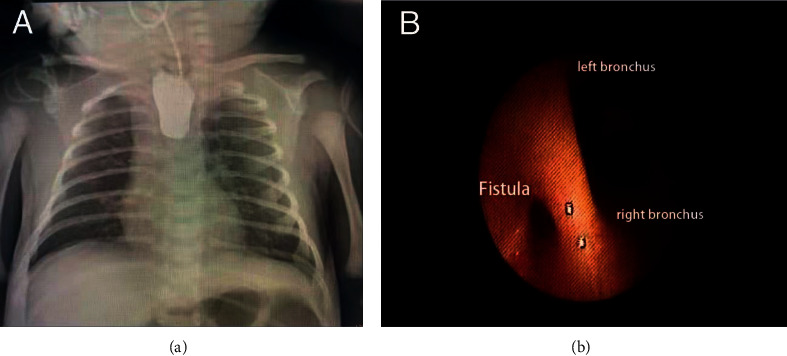
The contrast instilled in the esophageal pouch (a) and a fistula posterior to the left and right main bronchi (b).

A 41-week gestational age male neonate (2.77 kg) was diagnosed with anorectal atresia and admitted to the neonatal intensive care unit (NICU) for further evaluation of anal atresia. However, inserting a nasogastric tube for gastric decompression was difficult, and a chest radiograph demonstrated a proximal blind esophageal pouch and gastric distention (Figure 1(a)). Combined with the clinical symptoms of coughing and vomiting after the initial attempt of feeding, esophageal atresia (EA) associated with tracheoesophageal fistula (TEF) (EA/TEF) was diagnosed. Therefore, TEF repair and colostomy surgery were scheduled on postnatal day 3.

Upon arriving in the operating room, standard American Society of Anesthesiologists monitors were applied. The infant had rales on both sides of the lungs with a distended abdomen. 5 mg methylprednisolone and 0.1 mg atropine were given. Inhalational induction via facemask with sevoflurane was started with 1% and gradually increased (increment of 0.5% every 5 min) up to 3% while maintaining spontaneous ventilation. During the induction, we supplemented 3 mcg/kg fentanyl in increments of 1 *μ*g/kg.

After the infant's response to the jaw-thrust maneuver disappeared, we inserted a laryngeal mask airway (LMA) (size 1, Hisern, China). Simultaneously, we infused remifentanil (0.1 *μ*g/kg/min) and dexmedetomidine (0.5 *μ*g/kg/h) and sprayed 1% lidocaine over the glottis under the guidance of a flexible bronchoscope (2.8 mm, Olympus). Then, we inserted the flexible bronchoscope through the LMA into the trachea. The airway anatomy was evaluated and fistula between the trachea and esophagus was identified (Figure 1(b)). Then, we removed the LMA, and a bronchial blocker (5Fr, Wellead, China) was inserted into the trachea under direct laryngoscopy. The LMA was re-inserted alongside the bronchial blocker, followed by the bronchoscope insertion described above. With the assistance of the bronchoscope, the bronchial blocker was advanced into the fistula and cuff inflated. The blockade of the fistula was confirmed by absence of gurgling sounds over the stomach on auscultation during positive pressure ventilation by gently pressuring the breathing bag. Finally, we removed the LMA and intubated the trachea with a 3 mm cuffed tracheal tube secured at a depth of 9 cm from the tip. We re-confirmed the blockade of the fistula after tracheal intubation at the airway pressure of 25 cm H_2_O by auscultation over the stomach. The infant continued breathing spontaneously during the whole bronchoscopy and blocker insertion period, which avoided further gastric insufflation and pulmonary aspiration.

After securing the airway, we monitored the invasive arterial pressure via the radial artery. The neuromuscular block was achieved with cis-atracurium to facilitate surgical decompression for anorectal atresia. The lungs were ventilated at the peak airway pressure of 20 cm H_2_O and the rate of 35 breaths per minute.

Surgical decompression was first performed. The infant underwent colostomy in the supine position. Then, the newborn was placed in left lateral position for thoracotomy. The bronchial blocker helped the surgeon identify the fistula by palpation during the surgical procedure. Before ligation of the fistula, the blocker was deflated and withdrawn. The surgical procedure was completed uneventfully. The infant was transferred back to NICU with intubation and sedation. He was ventilated for three more days before he was extubated.

## 3. Discussion

Esophageal atresia (EA) with tracheoesophageal fistula (TEF) is a common congenital airway anomaly. It often occurs associated with other congenital disabilities such as VACTERL—vertebral defects, anal atresia, cardiac, trachea-esophageal fistula, and renal and limb defects [2, 3]. Among these associated birth anomalies, anorectal anomalies are not uncommon, with an incidence rate of 14% [2]. Due to the air leakage, gastrointestinal distension leads to progressive diaphragmatic elevation and can progress to respiratory failure. It is essential to block the fistula and reduce intra-abdominal pressure under anesthesia before proceeding for surgery.

In our case, we have shown that flexible bronchoscopy insertion through the laryngeal mask with spontaneous breathing would be simpler and safer than rigid bronchoscopy or endotracheal tube beyond the fistula for airway management of EA/TEF with anal atresia. The flexible bronchoscopy insertion through the laryngeal mask can examine the entire airway while preserving spontaneous ventilation. On the other hand, one may miss the higher fistula if bronchoscopy is done through the endotracheal tube. Kosloske et al. [4] suggested that bronchoscopy examination should be considered for all infants with EA/TEF to locate the fistula and other airway anomalies. Deanovic et al. [5] demonstrated that flexible bronchoscopy is easier to maneuver and can be repeated as necessary, compared with rigid bronchoscopy. In addition, Fogarty balloon or bronchial blockers can be placed via flexible bronchoscopy to block the fistula [6]. In our case, placing a bronchial blocker under flexible bronchoscopy guidance successfully occluded the fistula. In the event of fistula occlusion failure, such as the wrong size, the blocker's central lumen can still provide a route for gastric decompression.

Additionally, we performed flexible bronchoscopy via an LMA to ensure the patency of the airway during airway manipulation. Chou et al. [7] performed flexible bronchoscopy insertion via an endotracheal tube to check the fistula site. They believed that their patients could tolerate the flexible bronchoscopy procedure well. As we know, the laryngeal mask is an airway instrument placed above the glottis. Flexible bronchoscopy insertion via a laryngeal mask can inspect the whole trachea and prevent the occurrence of deoxygenation. As pointed out by Fraser et al. [8], they successfully used the laryngeal mask airway for transferring the infant with type 3 EA/TEF. Therefore, the use of a laryngeal mask in these patients is safe. It is worth mentioning that before fistula occlusion, the spontaneous breathing of the infant should be preserved, and small positive airway pressure (CPAP) ventilation has been shown to be safe for patients with EA/TEF before the fistula ligation. However, our infant with EA/TEF and anal atresia had more gastrointestinal distension and reflux risk. We believe that spontaneous breathing is safer for our patients.

In conclusion, using advanced airway management techniques in neonates with EA/TEF and anal atresia could improve clinical outcomes. The use of bronchial blocker occluding the fistula under the guidance of the bronchoscopy while maintaining the spontaneous breathing through laryngeal mask airway can be a preferred technique of anesthesia management for neonates of EA/TEF with anal atresia.

## Data Availability

All data generated or analyzed during this study are included within the article.
